# Patients’ Main Concerns About Having a Sibling Stem Cell Donor – A Grounded Theory Study

**DOI:** 10.2174/1874434601812010046

**Published:** 2018-03-30

**Authors:** Annika M Kisch, Anna Forsberg

**Affiliations:** 1 Department of Haematology, Skåne University hospital, S-221 85 Lund, Sweden; 2 Institute of Health Sciences, Lund University, Lund, Sweden; 3 Department of Thoracic Surgery, Lund University, Skåne University Hospital, S-221 85 Lund, Sweden

**Keywords:** Allogeneic haematopoietic stem cell transplantation, Sibling donor, HSCT, Interviews, Qualitative study, Grounded Theory

## Abstract

**Background:**

There is limited knowledge about the perspective of patients undergoing allogeneic haematopoietic stem cell transplantation (HSCT) about having a sibling as donor. It is essential to understand the main concerns of stem cell recipients in order to enable nurses to provide person-centred care.

**Objectives:**

The study aim was to explore patients’ main concerns about having a sibling stem cell donor and how the patients handle them, from immediately before until one year after transplantation.

**Methods:**

Twenty-eight interviews were performed prospectively during one year with ten adult sibling stem cell recipients with a mean age of 52 years (range 19-68 years). The interviews were analyzed by the Grounded Theory method.

**Results:**

The core category Recompensation summarises the process in the generated grounded theory including the three main categories; *Invest*, *Compensate* and *Celebrate*. Recompensation is defined as a lasting compensation given by the recipient to the sibling donor for the loss or harm suffered or effort made. The sense of having to reward, protect, appreciate, maintain peace and work on the relationship with the sibling donor at the same time as having to accept a serious illness, cope with their situation and promote their own recovery is strenuous for the recipients.

**Conclusion:**

The main concern for stem cell recipients during their first post-transplant year is to recompensate the sibling donor by investing, compensating and celebrating her/him. Although there is a positive aspect of recompensation, it can also imply pressure and guilt.

## 1 INTRODUCTION

This study was performed to gain deep knowledge and understanding about the perspective of patients undergoing allogeneic haematopoietic stem cell transplantation (HSCT) with a sibling donor. It is essential to understand the main concerns of stem cell recipients in order to enable health professionals to provide person-centred care.

Allogeneic HSCT is a well**-**established treatment that offers a potential cure for patients with haematological malignancies. One essential prerequisite for HSCT is finding a donor with a reasonably close Human leukocyte antigen (HLA) match to increase the patient’s chances of survival [1]. The donation of haematopoietic stem cells is performed either by peripheral blood stem cell collection (PBSC), Bone marrow harvest or by cord blood collection. Around two-thirds of all HSCTs are performed with stem cells from unrelated registry donors and one third with cells from sibling donors. In Sweden, around 280 HSCTs are performed every year. In Europe, the annual performance rate is more than 15,000 [2] and worldwide over 30,000 [3]. The one-year recipient survival rate is 70-80% [4].

Although HSCT is established as a successful treatment for haematological malignancies, there is a significant risk of acute complications, Late side effects and mortality [5, 6]. The most common and transient side effects from stem cell donation are fatigue, headache, bone pain, muscle pain and nausea [7-9]. Major complications due to stem cell donation are rare, but deep vein thrombosis, splenic rupture and cardiac arrest have been reported [10, 11].

Only one study has truly explored patients’ experiences of having a sibling donor in detail [12]. That study revealed that immediately before HSCT patients experience being in a complex situation characterised by a variety of emotions and thoughts, including concerns about the sibling donor and other relatives.

The sibling donors’ situation has also received little attention. These donors are in a vulnerable situation and both negative and positive experiences are reported, such as anxiety, pain, guilt, happiness, an increased sense of self-worth and pride [13, 14]. A recent grounded theory study on adult sibling donors describes that being a sibling donor means doing what you have to do to fulfil your duty as a sibling in order to try to save the life of a seriously ill brother or sister. The sibling donors’ efforts were summarised in a process with three main categories; Prepare, Promote and Preserve. A clear path of transition leading to fulfilment was revealed, starting before donation and continuing for one year afterward, during which the relationship between the siblings was strengthened by the donation process [15]. It is obvious that both patients and their sibling donors have different relations with family members and friends who are often engaged in or affected by the transplantation and donation, resulting in a social process of change. Being transplanted with stem cells from a sibling donor probably affects the patient’s life situation, thus, knowledge about the patients’ social situation after receiving stem cells from a sibling donor is needed to enable transplant nurses to provide person-centred care and support. Therefore, the study aim was to explore patients’ main concerns about having a sibling stem cell donor and how the patients handle them, from immediately before until one year after transplantation.

## 2 MATERIALS AND METHODS

The starting point for this study was patients’ main concerns about having a sibling stem cell donor from immediately before HSCT until one year afterward. Grounded Theory (GT) according to Charmaz [16] was the qualitative method chosen. The inductive constructivist approach made it possible to theorise on the informants’ interpretations obtained from the interviews. The authors have extensive pre-understanding from the field of transplantation. One of the authors has comprehensive nursing experience from HSC recipients and their donors, while the other author’s experience is derived from the care of recipients of solid organs and their donors. The interviews and the analysis led to a deeper knowledge and understanding of the recipients’ main concerns from before until one year after the transplantation, and how the patients handled them.

### 2.1 Ethical Considerations in Research

The approval of the study was made by the Regional Ethical Review Board of Southern Sweden (Dnr 541/2007) and performed in accordance with the Declaration of Helsinki [17]. The potential risks to the recipients connected with the interviews were judged to be small. However, since there was a risk that the interviews could cause emotional reactions, they were offered the possibility of psychosocial support at the hospital. The recipients were informed, verbally and in writing that their participation was voluntary and that they had the right to withdraw from the study at any time without giving any reason. The first author (AK) obtained the recipients’ written informed consent before the first interview took place.

### 2.2 Selection and Recruitment

Patients planned for HSCT with a sibling donor at a University Hospital in Sweden between March 2011 and December 2012 were consecutively asked to participate in the study. All recipients and their sibling donors were ≥ 18 years and had good ability in understanding and speaking Swedish. Ten patients who met the inclusion criteria were asked to participate in the study and all ten agreed to do so. At the recipients’ appointment for medical investigation and information pre-transplantation, they were informed and asked about participation in the study by the first author (AK). We decided from the start to follow the included ten participants during one year. Thus, the methodological step involving true theoretical selection was abandoned. Instead, we ensured that the sample would reflect clinical reality. After agreement to participate, none of the recipients were excluded or decided to withdraw from the study. All donors of the recipients participated in a corresponding interview study [15].

### 2.3 Data Collection

The method chosen for data collection was face-to-face interviews performed by the first author (AK) at a time and location decided by the informants. The first author (AK) is a nurse specialist and has extensive experience from nursing experience from recipients of stem cells and from stem cell donor care, but did not take part in the care of the informants in this study. Altogether, 28 interviews were carried out: immediately before transplantation (eight informants were interviewed on the day of admission, One informant the day prior to admission and one informant eleven days before admission), and three months and one year after HSCT. One recipient only participated in the first interview as she, unfortunately, died due to complications from the transplantation less than three months afterward. All interviews began with the open question: “Can you tell me about your thoughts and feelings when you became aware that you needed a stem cell donor for transplantation?” followed by: “What did you feel and think when you were told that a sibling could become your donor?” and “Can you please tell me now, three months/one year after transplantation, what being transplanted with stem cells from your sister/brother was like?” Additional questions were asked to make the recipients elaborate their answers about their experiences and thoughts about having a sibling donor, and also to extend the answers given in prior interviews. The interviews were digitally recorded and lasted between 14 and 121 minutes (median 53 min). After finalizing each interview they were transcribed verbatim.

### 2.4 Data Analysis

A search of the literature revealed that no study had been performed within this particular context, which is in line with the recommendations of Hallberg [18] and Glaser [19]. The interviews in this study and in the study of the sibling donors [15] were performed simultaneously by Grounded Theory according to Charmaz [16]. A detailed description of the analysis method was given in Kisch & Forsberg, 2017 [15].

## 3 RESULT

### 3.1 Informant Characteristics

A total of ten adult patients undergoing HSCT, four men, and six women, participated in the study. Their mean age at the time of the first interview was 52 years (19-68 years). Demographics and characteristics of the recipients are presented in Table **1**. The mean age of the sibling donors was 49 years (26-66 years). The gender constellation was: male donor to male patient (3), male donor to female patient (2), female donor to male patient (1), female donor to female patient (4).

### 3.2 Recompensation

The essence of the results derived from the grounded theory analysis of the interviews shows the core category Recompensation and the three main categories; *Invest*, *Compensate* and *Celebrate.* Recompensation is defined as a lasting compensation given by the recipient to the sibling donor for the loss or harm suffered or effort made, *i.e.*, the stem cell donation.

**Fig. (1):**
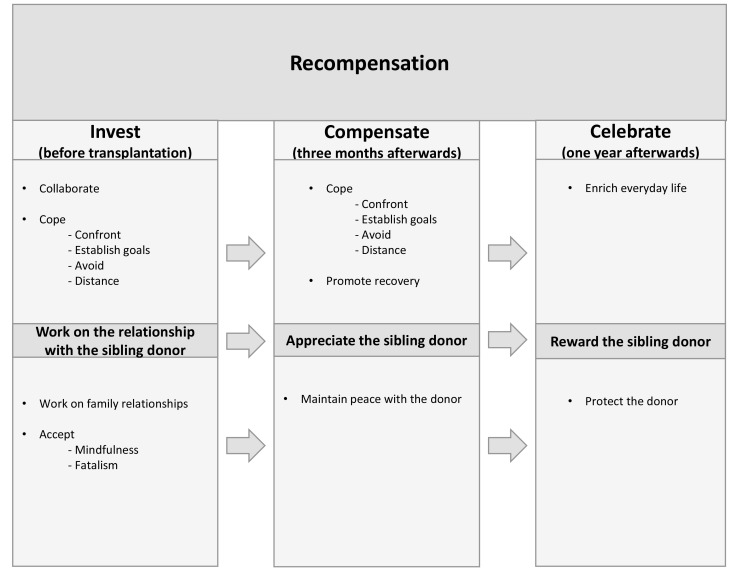
The grounded theory of recompensation among patients transplanted with stem cells from a sibling donor, derived from the analysis of the interviews, involves three distinct phases, before donation - *Invest*, three months afterwards *- Compensate* and 12 months afterwards *- Celebrate*. Recompensation is defined as a lasting compensation given by the recipient to the sibling donor for the loss or harm suffered or effort made, *i.e.*, the stem cell donation. Investment is the starting point for the recipient’s recompensation for the sibling donor. The efforts made in this phase are investments in the transplantation project, *i.e.*, the recipient’s survival. Compensation three months after the transplantation means the start of the pay-back to the sibling donor by a two-fold approach: firstly, coping with the situation and recovering and secondly, thanking the donor and protecting the relationship with her/him. One year after transplantation celebration means paying tribute to the donor and oneself and keeping the stem cells working. In a worst-case scenario it involves protecting the donor from the knowledge that the recipient is suffering and possibly facing death.

For the recipients, the most important aspect was to recover from the serious illness. In order for this to happen, they were painfully aware of the need for a donor, while at the same time they did not wish to harm anyone. Receiving stem cells from a sibling implied a sense of security for the recipient, *i.e.*, knowing the origin of the stem cells, the donor’s previous lifestyle and what kind of person the donor is. The alternative, *i.e.*, receiving stem cells from a stranger, a registry donor, meant a great sense of uncertainty about the origin of the stem cells and the unfamiliar donor’s life and personality. The consequence of donation from a stranger was perceived as difficult to grasp. In one way receiving stem cells from an unrelated donor would be easier because the sense of guilt towards the donor would be less. However, the recipients preferred a sibling donor to an unrelated one, Even though, it meant a sense of guilt about the sibling. This sense of guilt entailed a need to compensate the donor for her/his effort, which became evident by the recipients’ recompensation. The recompensation was, manifested in taking great responsibility for her/his well-being and possible pain, harm and fear, which started even before the donation and transplantation. The process of recompensation from before until one year after transplantation clearly demonstrates the recipients’ efforts to protect the donor and show their gratitude, even though they are unable to alleviate the donor’s suffering. Furthermore, the three main categories comprise nine sub-categories illustrating the strategies used by the recipients to recompensate, (Fig. **1**). The core of the process is recompensation towards the sibling donor, while the driving force is the balance between guilt and compensation.

Working on the relationship with the sibling donor was an important strategy for all of the recipients throughout the first year, from immediately before until one year after HSCT, irrespective of the closeness in relation with the sibling donor. All recipients made efforts, regardless of their capacity and situation, to keep in contact with the sibling donor in the year following transplantation and wished to preserve the relationship. When the relationship was good their efforts concerned maintaining the proximity and companionship. When the quality of the relationship was poor or even non-existent the informants tried to increase proximity in various ways or at least make the donor aware of their gratitude by enlisting the help of other family members to convey their message. Thus, the recipients hoped for survival and possibly better health. Regardless of the level of proximity, geographical distance or quality of the relationship they tried to recompensate their sibling for her/his loss, harm or effort due to the donation. The main categories and sub-categories will be presented in bold italics illustrating the strategies used by the recipients during the first year.

### 3.3 Invest (Pre-Transplantation)

Investment is the starting point for the recipient’s recompensation towards the sibling donor. The efforts made in this phase were intended as investments in the transplantation project, *i.e.*, the project that would ensure the recipient’s survival. Thus, if the recipient prepared her/himself, coped and worked on various relationships, in particular, the sibling relationship, she/he thereby optimized the circumstances so that there would be less harm to compensate afterward.

The interviews revealed that for the recipients, the pre-transplantation phase lasted from the time the donor was identified until the planned donation. To *collaborate* was a way of investing in themselves by self-optimization. This involved having a positive attitude, preparing themselves practically, socially and professionally and taking care of themselves, *e.g.*, eating well, exercising and not smoking. They also chose to trust and rely on health care and healthcare professionals. Some donors expressed this as: helping the healthcare professionals to help them by making themselves deserving of the donation.

“Being away from home for such a long time and not knowing if I would ever come home again, or what shape I would be in…it demands a lot of long-term planning.” (female recipient, 62 years)

“try to be strong, try to eat even when I have no appetite and try to drink even if I can’t stand it. Everything to help you cure me, so to speak.” (female recipient, 68 years)

Before transplantation, the recipients made different attempts to ***cope*** in various ways to invest in the situation. They adopted different coping strategies and played either an active role by establishing goals for the future and confronting the situation, *e.g.*, by reading, planning, thinking, discussing, informing relatives and friends, or a passive role by avoiding and distancing themselves from the situation.

“Now I have these goals… things that will happen during this year, that I know I want to be a part of. First, there is the sailing and then I will become a grandfather.” (male recipient, 57 years)

“My family knows. But I haven’t told my friends or colleagues at work… There are too many questions, they speculate and I’m not interested. They can’t help me.” (female recipient, 62 years)

A common approach among all informants, regardless of the closeness of the relationship, was to *work on the relationship with the sibling donor* to ensure the donation. This included taking responsibility for the relationship by informing themselves about the donation procedure, increasing the amount of contact with the sibling donor and paying extra attention to her/him. The recipients trusted the donor to complete the donation and some even offered the sibling donor an opportunity to withdraw.

“They said that she would experience the bone marrow harvest like being kicked in the back or on the behind by a horse. But we haven’t told her that. So she’s probably not aware of what is coming. I am worried because she is a bit worried about it and when she is extra worried I chat with her every day.” (female recipient, 19 years)

Another way to ensure the donation was to ***work on family relationships*** by taking responsibility for the relationship between various members of the family. This was done by, for example, involving the family in the transplantation procedure, listening to and talking more with different family members than before and playing down the risks of the transplantation to reduce their worries. The aim of this strategy was to cause no conflicts, thereby preventing the risk of doubt or regrets on the part of the donor, her/his spouse/partner or significant others. The investment was all about not jeopardizing the “transplantation project” or what was at stake.

”My focus is on his wife and children. I feel I need to talk more with my sister in law… I want to acknowledge the fact that she is making an effort too.” (male recipient, 39 years)

A part of the investment was also to *accept* the serious illness and that the transplantation would be hard and strenuous. All recipients managed to practice the art of mindfulness and took one day at a time, reasoning that whatever happens will happen. However, at the same time as they expressed that they mainly relied on faith in a fatalistic manner, They directed all their efforts to ensure the donation from their sibling in a very concrete way.

“I have to be very enthusiastic and very grateful… I simply have to put her first…. I need to keep her in a very good mood now.” (female recipient, 62 years)

### 3.4 Compensate (Three Months Post-Transplantation)

Compensation, defined as repaying the sibling donor, started three months after transplantation by means of a two-fold approach: firstly, coping with the situation and recovering and secondly, thanking the donor and protecting the relationship with her/him. To *cope* three months after transplantation meant managing the situation of being recently transplanted with stem cells from a sibling donor and having to recover. Although the sequence in which the coping strategies were employed differed between individuals, they all made use of the same strategies at some stage of the period from before transplantation until three months afterward. Either the recipients played an active role by confronting the situation, processing what they had gone through, prioritizing themselves and establishing goals for the future, or a passive role by avoiding and distancing themselves by not talking or thinking about the situation very much.

“I try to avoid thinking about it as much as possible and do as many things as possible to be active and on the go.” (male recipient, 39 years)

“I make no plans but simply try to take each day as it comes.” (female recipient, 62 years)

During this phase, they *promote recovery* in various ways, depending on the individual and on the outcome of the transplantation. They accepted the situation and their level of well-being in order not to disturb the recovery process. Many of the recipients focused on the new stem cells and some even talked to the cells and ate sweets that the donor liked to feed the stem cells in a way they were used to.

“This might sound weird, but I talk to my new stem cells every day. I have tried, this is a part of my pep talk. Therefore we, my stem cells and I, will make this work. I tell them that they are welcome to my body to remove my sick cells.” (female recipient, 62 years)

The recipients who were feeling well enjoyed being out of the hospital, tried to create new routines in their everyday life and started to plan their future. Those who were not feeling so well tried to appreciate the positive aspects of their situation and did their best not to show their malaise to family and friends.

” I try to eat fatty food, using butter and cream, eating carbohydrates and sugar. I try to keep to that diet and eat as much as I can. I simply try to do my best. This is my only option. Simply bite the bullet and look happy.” (male recipient, 57 years)

An essential strategy three months post-donation was to *appreciate the sibling donor*. They thanked their sibling donor by verbally expressing their gratefulness, by not showing any impatience in their contact with the donor and telling her/him what a great contribution she/he has made. Most of the recipients were planning some kind of reward for the donor, *e.g.*, offer a holiday or a visit to the theatre.

“I will try to compensate if he suggests something. He would love to travel with me sometime. So, I feel I will do it for him, if that is his wish.” (female recipient, 68 years)

It was very obvious that the recipients worked to *maintain peace with the donor.* They thanked the sibling donor for the gift by ensuring that no conflict arose with the donor or her/his family. Peace was also maintained by increased contact with the sibling donor and her/his family, in the course of which the recipient treated them with greater understanding and patience.

“Maybe you’re… more careful to never show impatience when he phones me. Sometimes I have been very tired and he wants to talk forever…” (female recipient, 68 years)

### 3.5 Celebrate (One-Year Post-Transplantation)

Celebration meant paying tribute to the donor and to oneself, as well as keeping the stem cells working. The celebration involved enriching everyday life, rewarding and protecting the donor. The ways of celebrating depended to a large extent on the recipient’s well-being. One year after transplantation all recipients had changed their perspective on life and the majority took advantage of the time they had been given. They tried to *enrich everyday life* and to do the things they enjoyed, but it was dependent on their illness burden, which was rather extensive for some of the informants. One recipient who was doing very well decided to retire from work to have more time for joy. She allowed herself various new things and made plans to travel. Those who experienced health lived a good life and made plans for the future.

“I am content that I have retired. To do things I want to do, living a good life. As I say, it is better to live in the present, spend your money. I travel wherever I want and do all the shopping I want now.” (female recipient, 62 years)

Those who suffered from severe illness due to complications and set-backs tried to make the best of everyday life by undertaking light activities at home, *e.g.*, painting pictures, sewing or simply sitting on the balcony. This served as a way of paying tribute to oneself and even suppressed the fact that they were doing badly. However, one recipient had no strategies whatsoever one year after transplantation and experienced only suffering and disappointment. For this particular recipient, there was no celebration at all.

“I can say one thing. If I had known that I would feel this poorly I would never have accepted the treatment. It is not worth it.” (female recipient, 62 years)

The recipients who experienced health one year after the transplantation used different strategies to *reward the sibling donor*. They made plans to reward the donor and her/his partner by, for example, celebrating the first anniversary of the donation and transplantation.

“I had this huge celebration on my birthday and E (the donor) was there. He was sort of a prince and that was simply right. The donor should be in the spotlight and one should not forget that, it is extremely important.” (female recipient, 68 years)

Appreciation was verbally expressed to the sibling donor by a simple ‘Thank you’, but also by describing how the good quality of the stem cells had contributed to excellent well-being. One recipient expressed that he will never ever be able to become angry with the sibling donor again. As the donor had saved his life he will be forever indebted to him.

“I will probably not be pissed off with him, at least not for a while. Because it feels like no matter what he does it is a mere trifle in the context.” (male recipient, 39 years)

The recipients who suffered from severe illness lacked conditions or had few opportunities to reward the donor. These recipients suffered a great deal one year after transplantation and realised that they would probably not survive. Thus, they prepared themselves in different ways for their death, while at the same time trying to *protect the sibling donor* from knowing. They avoided talking to the donor about their malaise and attempted to conceal how they were doing.

“Unfortunately, I haven’t had the strength to keep in contact lately. I mostly use text messages or the computer, or suchlike.” (male recipient, 61 years)

The wish to protect the donor was handled in different ways by the recipients. One example is the recipient who eventually needed a second donation from her sister. However, the sister was pregnant and therefore could not donate again. The recipient did not want to burden the donor and also felt guilty due to being unable to rejoice about her sister’s pregnancy. Another example was the recipient who was suffering from severe chronic GvHD and did not want to burden the donor with the knowledge of her condition. At the same time, she experienced a sense of guilt because she was aware that the sibling donor had suffered during the donation procedure.

“ I felt very selfish…. no, you can’t be pregnant. I felt like I ruined her happiness about the pregnancy. I needed her help and she wanted to start a family.” (female recipient, 19 years)

In conclusion, throughout the first year after stem cell transplantation, in sickness and in health, the stem cell recipients strived to recompensate their sibling donor for the loss, harm or effort caused by the donation, while at the same time coping with their own suffering and losses.

## 4 DISCUSSION

### 4.1 Methodological Considerations

No prospective grounded theory studies of recipients of stem cells from sibling donors have been previously performed, as far as we know. We chose to use the criteria for rigour in Grounded Theory studies by Charmaz [16]. Credibility was reached by performing line-by-line coding of the content in the transcribed interviews that indicated important parts corresponding to the study aim, and by inserting illustrative quotes in the Result section of the manuscript. The main and subcategories reveal that recipients of stem cells from a sibling donor deal with a great variety of experiences from immediately before until one year after HSCT.

To confirm and optimise the result, the codes, sub and the main categories were all along the analysis checked against the transcripts of the interviews. We believe that the result from this study is relevant to the recipients of stem cells from sibling donors in similar contexts as in this study; *i.e.* with similar health care as in Western countries. This generated Grounded Theory has to be further tested to secure the applicability to recipients of living organ donors, *e.g.*, kidney donors.

One limitation of this study is the possibility of transferability since this study was conducted in only one country, Sweden, with only Swedish speaking recipients. The study sample is small; however, it is representative of adult recipients of stem cells from adult sibling donors; the median age of 61 years and about the same number of women and men. By choosing in this context a unique prospective design we did not fulfil the true methodological step where sampling is performed until theoretical saturation. However, the 28 interviews enabled an in-depth understanding of the transplantation process.

### 4.2 Discussion of the Findings

To the best of our knowledge, this is the first study of the main concerns of recipients transplanted with stem cells from a sibling donor, making it a unique contribution to the field of stem cell transplantation. This study and the recent grounded theory study on adult sibling donors’ main concerns [15] show the processes of both recipients and sibling donors, as well as what happens in their social contexts. Interesting findings are the completely different main concerns of the sibling donors and the recipients during the donation and transplantation process. For the sibling donors, the main concern was to fulfil their duty as a sibling by doing what they considered necessary in order to, if possible, save the life of a seriously ill brother or sister. The sibling donors did not wish to be thanked or celebrated, while for the recipients, the core of the process was recompensation the sibling donor by celebrating the donation and paying tribute to the donor. It is important for nurses to be aware of these two different processes of change driven by different main concerns, as they may give rise to a complicated relational process between two siblings. The donors tried to prepare, promote, preserve and minimize their effort as a donor, while the recipients attempted to invest, compensate and celebrate, which could cause paradoxical communication and sibling relationships. This has never been studied before, thus making this knowledge new. Based on our clinical knowledge, we argue that healthcare professionals are not aware of these two different processes of change and different main concerns, and that having a sibling as donor might not only be experienced as something positive but also as a burden. This study, together with the grounded theory study of sibling donors, contributes knowledge of both perspectives to the field of stem cell transplantation; *i.e.*, that of the recipient and the donor.

The quality of life (QoL) of patients treated with HSCT has been explored in several previous studies [6, 20-22] mainly showing that QoL deteriorates immediately after HSCT and stabilises or improves after three months [21-24]. Emotional well-being is usually most impaired before and immediately after HSCT, but improves over [23, 24]. The data regarding changes in the physical and social well-being are conflicting. One study on QoL from before until one year after HSCT reveals that patients’ physical and social/family well-being deteriorates between baseline and the 12-month follow-up, while emotional well-being improved [20]. One main factor associated with deteriorating social well-being over time was found to be transplantation with stem cells from a sibling donor (a.a). In this study, we explored patients’ main concerns about having a sibling stem cell donor. The result of patients’ recompensation, defined as a lasting compensation given by the recipient to the sibling donor for the loss or harm suffered or effort made, *i.e.*, the stem cell donation, can partly explain the recipients’ decreased social well-being over time. For many recipients, the sense that they have to reward, protect, appreciate, maintain peace with and work on the relationship with the sibling donor, while at the same time having to accept a serious illness, cope with their situation, promote their own recovery enrich their everyday life is most certainly strenuous. In addition, the result shows that recipients of stem cells from a sibling donor experience that they have to work on family relationships, especially the relationship with the sibling donor. Therefore, one assumption is that in some ways it might be easier for the recipient to receive stem cells from an anonymous registry donor than from a sibling donor. The theoretical framework of recompensation among patients transplanted with stem cells from a sibling donor may be of value in supporting patients undergoing allogeneic haematopoietic stem cell transplantation (HSCT) with a sibling donor and to enable stem cell transplant nurses to facilitate person centred caring interventions.

## CONCLUSION

1. The main concern for stem cell recipients during their first post-transplant year is to recompensate the sibling donor by investing, compensating and celebrating her/him.
2. Although there is a positive aspect of recompensation, it can also imply pressure and guilt.

### CLINICAL IMPLICATION

This knowledge of the perspectives of both the recipient and the donor could allow transplant professionals to consider trying to influence the situation. One way of doing this might be to organize a formal occasion for celebration and closure. This could prevent the long-term pressure of recompensation among recipients suffering from various side-effects and complications, as they already have enough problems to handle. Patient information to recipients could include a paragraph stating that the donor usually does not wish to be celebrated, which would help to ease the recipients’ burden.

## Figures and Tables

**Table 1:** Demographics and characteristics of the recipients.

Characteristics	n =10 *n*
*Age, years* Mean (range)	52 (19-68)
*Sex* Female Male	6 4
*Marital status* Married/living together Single	6 4
*Diagnosis* AML NHL MPD ALL CML SAA	3 2 2 1 1 1
*Stem cell source* PBSC BM	9 1
*Gender of donor* Female Male	5 5
*Contact with donor* Frequent contact Occasional/no contact	6 4
*Recipient status three months post-donation* CR, doing well Severe GvHD Deceased *Recipient status one year post-donation* CR, doing well Severe GvHD Deceased	4 5 1 4 5 1

ALL = Acute lymphoblastic leukaemia; AML = Acute myeloid leukaemia; BM = Bone marrow; CML = Chronic myeloid leukaemia; CR = Complete Remission; GvHD = Graft versus Host Disease; MPD = Myeloproliferative disease; NHL = Non-Hodgkin lymphoma; PBSC = Peripheral Blood Stem Cells; SAA = Severe aplastic anaemia
